# Research Trends on Motivation and Job Satisfaction: A Scientometric Analysis

**DOI:** 10.12688/f1000research.165866.1

**Published:** 2025-07-17

**Authors:** Basudeb Jana, Bijayalaxmi Rautaray, Dillip K Swain, Chandrakanta Swain

**Affiliations:** 1 Library and Information Science, Kalinga Institute of Industrial Technology, Bhubaneswar, Odisha, India; 2 Director, Library & Head, Dept. of Library and Information Science, Kalinga Institute of Industrial Technology, Bhubaneswar, Odisha, India; 3 Faculty, Dept. of Library and Information Science, Kalinga Institute of Industrial Technology, Bhubaneswar, Odisha, India; 4 Library, Indian Institute of Management Raipur, Chhattisgarh, Raipur, India

**Keywords:** Scientometrics, Job satisfaction, Collaborative research, VOSviewer, Bibliometrics

## Abstract

**Background:**

This study explores the scientometric dimensions of publications related to “Job Satisfaction” and “Motivation” in the past 10 years by using the required analytical techniques for mapping the research output and metrics. Publication data on job satisfaction, ranging from 2014 to 2023, were extracted from Scopus.

**Methods:**

The types of records in the literature, including authors, nations, keywords, references, and citation counts, were studied using scientometric analysis. The keywords “Job Satisfaction” and “Motivation” were used to gather all data from the SCOPUS database as of January 10 ^th^, 2025. Both VosViewer and Biblioshiny were used to analyze the data. AScientometric method was used to determine the various parameters that fulfilled the research objectives.

**Results:**

The study finds that there has been an increasing trend of publications and their corresponding citations from the year 2014 to 2023 and the USA publications received the highest number of citations of 8350, followed by the Netherlands (4832 citations), and the United Kingdom (2595 citations).

**Conclusion:**

The evolution of publications on “Motivation” and “Job Satisfaction” from 2014 to 2023 showed a steady growth rate, despite annual variations over the previous ten years. Research on job satisfaction and motivation has advanced through the work of authors from the USA. We mapped the entire research structure and features of job satisfaction research over the past decade. This was accomplished by examining the research topics of motivation and the analysis revealed a gradual rise in scientific output on the subject of “Job Satisfaction” and “Motivation” from 2014 to 2023. Overall, the study’s findings show that research productivity in the Human Resource Management literature is increasing, which seems to be a healthy trend. The findings of this study add niceties to the corpus of existing literature.

## Introduction

Hertzberg posits that employees’ level of job satisfaction is truly influenced by both internal and external factors in their working ambience. Job satisfaction and motivation are related to each other; if one is affected, the other will also be affected. Few studies have been conducted on this topic. All studies emphasized the impact of employers on the satisfaction level of employees. Motivation is the inherent impulse to engage in actions necessary to achieve one’s objectives. Employees may be driven by various factors, including the work environment, financial compensation, and benefits for employees and staff members. Motivation may also stem from fear of termination, response, or humiliation among peers. Motivation can be defined as the process of exciting, inspiring, and encouraging employees to achieve their highest capacities. Motivation is a psychological concept that cannot be applied to employees. It originates intrinsically from personnel, as it reflects their motivation to perform tasks ( Francis, 2023). Employee motivation is considered the most important component of organizational goals and achievements since it enormously helps in providing fair returns on investments. Similarly, Job satisfaction is characterized as the degree of contentment of employees’ experiences regarding their employment. The impact of their work on their personal life, contentment with team members and management, and satisfaction with organizational rules are all covered in addition to their daily responsibilities. Many studies have been published on motivation and job satisfaction. However, the impact and significance of these studies have not yet been properly evaluated.

Therefore, we focused on the progression of scientific and scholarly communication concerning Motivation and Job Satisfaction in the last decade. Therefore, this study aims to analyze research trends exclusively in the domains of motivation and job satisfaction.

## Review of literature

Several significant studies on scientometric and citation analysis have been conducted by scholars globally. Scholars have used a variety of scientometic tools such as Biblioshiny, VOSviewer, and BibExcel to conduct scientometric analysis of literature on various topics **(** Wahid et al., 2023; Fernandes-Nascimento et al., 2023; Zheng et al., 2023; Yumnam et al., 2024; Feng, Wang, & Su, 2024; Klarin & Xiao, 2024; Nadanveettil et al., 2024). The use of VOSviewer software to analyze the global networks and co-occurrence of keywords was also found in the studies of Srivastava et al. (2021) and Samanta, Rautaray, & Swain (2023).

The findings of Vishnupriyan et al. (2023) provide insightful guidance for scholars of bibliometric studies to unfold different facets of research on a particular subject domain, including network analysis, research collaboration, and assorted facets. Sun et al. (2022) conducted a scientometric analysis of 507 articles retrieved from the Web of Science from 1996 to 2021 and used VOSviewer to visually map and analyze the development of transit-oriented development research. They found that documents with high co-citation strength in the four clusters had a significant impact on planning factors on transportation benefits.

Numerous investigations have been conducted to examine the scientific outputs of management and its sub fields. Syahid & Aghayani (2024) found that scientific output has increased exponentially in recent years, indicating that this increasingly strategic area has opportunities for further development.

Kannan, Kulandai, & Ramachandran (2024) in the bibliometric analysis revealed key factors associated with motivation, job satisfaction and retention of nurses in their respective working places. Moreover, the study significantly contributes to the understanding of nurse job satisfaction, retention dynamics, and assorted facets of banking on the findings of earlier studies.

Ryan & Deci (2000) conducted a review of scholarly publications spanning 23 years, and explored the impact of motivation on employee productivity. Wang & Asniza (2023) studied writing on motivation from the 21st century (2001–2022), reflected in the Web of Science and Scopus, and revealed patterns, citation frequency, and keyword co-occurrence using CiteSpace’s scientometric perspective.

Veerabbayi et al. (2023) identified the most influential articles, with social and practical implications, and pointed out the research gaps that demand comprehensive future studies that could investigate the intricate factors that relate to the motivation and productivity of different organizations. Alper (2025) studied the literature related to work motivation and productivity published from 1953 to 2024 and revealed the most productive countries, the most used keywords by authors, and trending topics among other significant facets.

Cvetkoska, Eftimov, and Kitanovikj (2025) conducted a bibliometric analysis to comprehend the links between corporate social responsibility and employee satisfaction and revealed that employees’ job satisfaction is largely associated with the company’s performance. Moreover, this study highlights the overlaps and connections between the two phenomena in human resource management (HRM).

Although there have been a few studies on this topic, the present study addresses the key bibliometric dimensions of motivation and job satisfaction in the corpus of recent publications during the last decade, which have not yet been explored.

### Objective of the study

This study sought to assess and offer a Scientometric analysis of the data obtained about the influence of motivation on job satisfaction in light of the preceding impacts. The primary objective of this study is to gain a thorough understanding of the relationship between motivation and job satisfaction by conducting a systematic review and data analysis of the body of existing research in the last decade and:

• To determine the most important sources and authors’ contributions to the fields of motivation and job satisfaction, as well as the annual growth of scientific research output and citation trends.
• To comprehend which nations are the most research productive countries.
• To identify the most prolific authors who have contributed significantly to the area of motivation and job satisfaction.
• To identify the major source journals those have published scholarly literature on job satisfaction and motivation.
• To identify highly cited Scopus indexed articles and their corresponding Google Scholar Citations.
• To identify the top keywords frequently used by authors in scholarly articles published in the area of job satisfaction and motivation.
• To find out the co-authorship network among countries.
• To comprehend co-citation network analysis among countries; and
• To ascertain the authors’ keyword analysis, the clustering of keywords, and co-authorship patterns.

## Methods

To meet the key objectives of this study, scholarly publications pertaining to job satisfaction and motivation were extracted from the online collections. Consequently, we obtained a list of scholarly works from the Scopus database.

The types of records in the literature, including authors, nations, keywords, references, andcitation counts, were studied using scientometric analysis.

The keywords “Job Satisfaction” and “Motivation” were used to gather all data from the SCOPUS database as of January 10 ^th^, 2025. Both VosViewer and Biblioshiny were used to analyze the data. A Scientometric method was used to determine the various parameters that fulfilled the research objectives.

**Figure 1.**
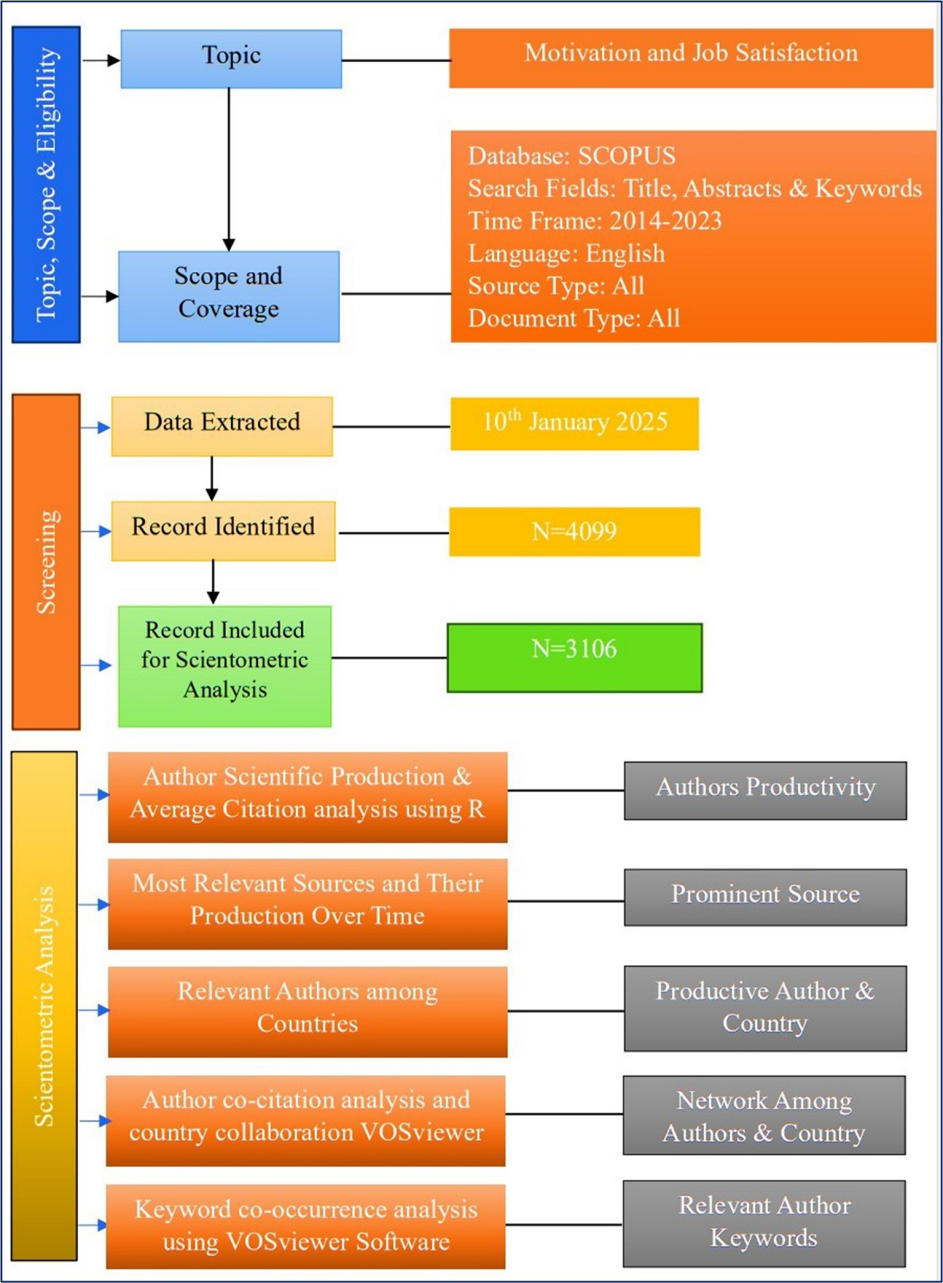
Research design.

When conducting a bibliometric study, it is necessary to have a research design as an essential component of the research methodology. Figure 1 shows how the research progressed using the step-by-step method.

### Data analysis

**Figure 2.**
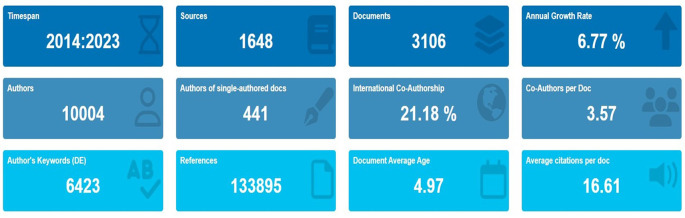
Key metrics information.

All types of documents published in the areas of motivation and job satisfactionfrom 2014 to 2023 were processed. The overall number of records was 3106 for the last 10 years. Moreover, the documents retrieved from Scopus carry the essential characteristics of scientific production over the years ranging from to 2014-2023. Figure 1 shows the complete information on scholarly production. The results indicate a total of 3106 documents retrieved during this period, with anannual growth rate of 6.77%, which indicates an excellent growth pattern that is partially identical to the findings of Bhargava & Ligade (2023). There are a total of 10004 authors, of which 441documents were found to be single-authored documents; over 43% of authors were published individually; the remaining authors were published as co-authors, and the co-authorship rate per document was 3.57. The total number of keywords in all documents is 6423, indicating that there are typically two keywords per document, similar to the findings of Tanko et al. (2023). The data is shown in Figure 2.

Table 1 lists the complete publication information for the retrieved documents.

**Table 1.** Key data.

Description	Results
Timespan	2014 to 2023
Sources (Journals, Books, etc)	1648
Documents	3106
Annual Growth Rate %	6.77
Document Average Age	4.97
Average citations per doc	16.61
References	133895
**Document Contents**	
Keywords Plus (ID)	5693
Author’s Keywords (DE)	6423
Authors	10004
Authors of single-authored docs	441
**Authors Collaboration**	
Single-authored docs	456
Co-Authors per Doc	3.57
International co-authorships %	21.18
**Document Types**	
Article	2592
Book	11
Book chapter	91
Conference paper	187
Conference review	7
Data paper	3
Editorial	19
Erratum	1
Letter	16
Note	41
Retracted	2
Review	131
Short survey	5

Table 1 shows that a total of 10004 authors from 1648 source journals and books produced 3106 documents from 2014 to 2023. Out of 3106 documents, 456 were found to be single-authored documents, while all other documents were found to be in the collaborative mode.

### Annual scientific production

**Figure 3.**
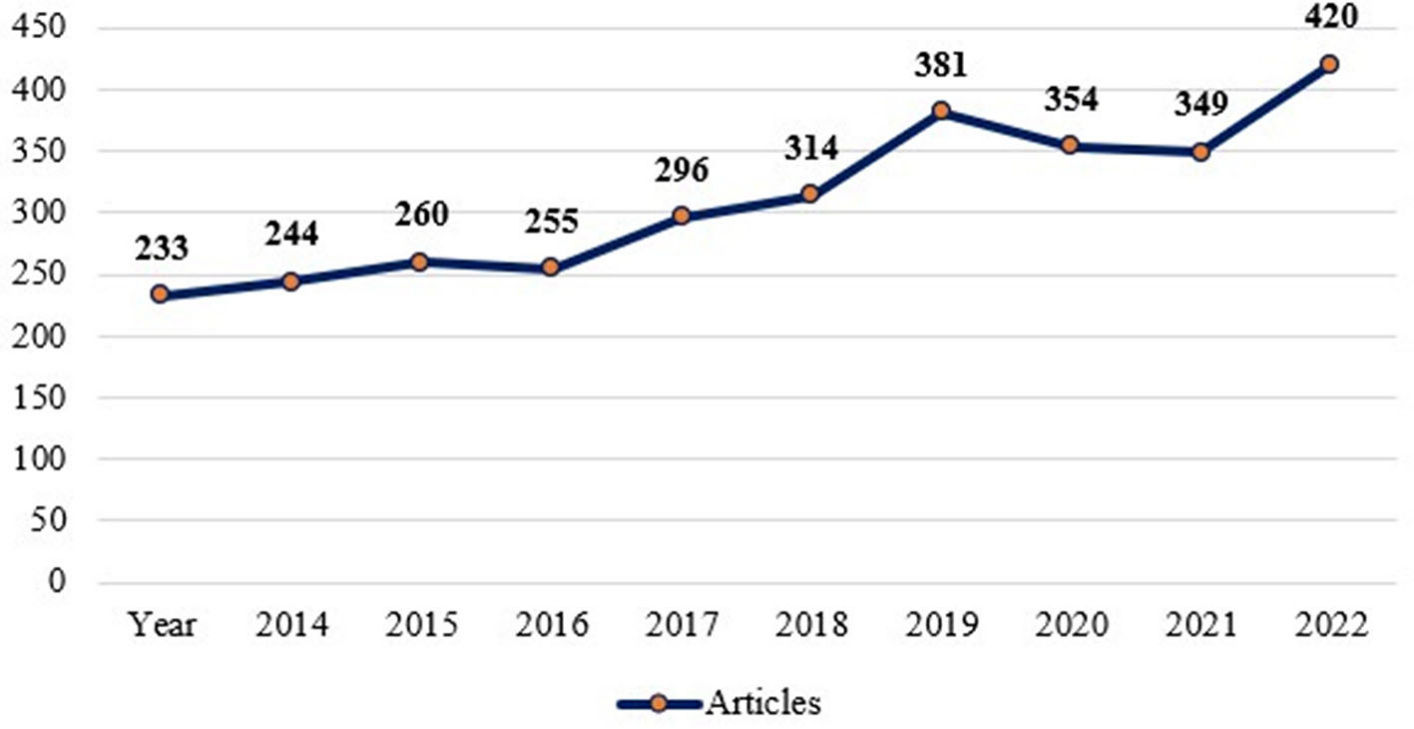
Annual scientific production.

The yearly scientific output of the “Motivation” and “Job Satisfaction” studies are shown in Figure 3. The amount of research produced each year in the areas of job satisfaction and motivation is increasing.

Table 2 provides a numerical description of the study.

**Table 2.** Annual scientific production.

Year	Articles	Year	Articles
2014	233	2019	314
2015	244	2020	381
2016	260	2021	354
2017	255	2022	349
2018	296	2023	420

Table 2 shows that the highest number of articles on motivation and job satisfaction were published in the year 2023 (420 articles) and the lowest number of articles were published in 2014 (233 articles). Though there has been a rising trends of published literature on motivation and job satisfaction, there were a little less publications in 2017 (255 publications) compared to that of its previous year in 2016 (260 publications). Similarly, the year 2022 (349 publications) are found a little less than that of its previous year 2021 (354 publications) which was again lesser than its previous year 2020 (381 documents).

### Citation trend

**Figure 4.**
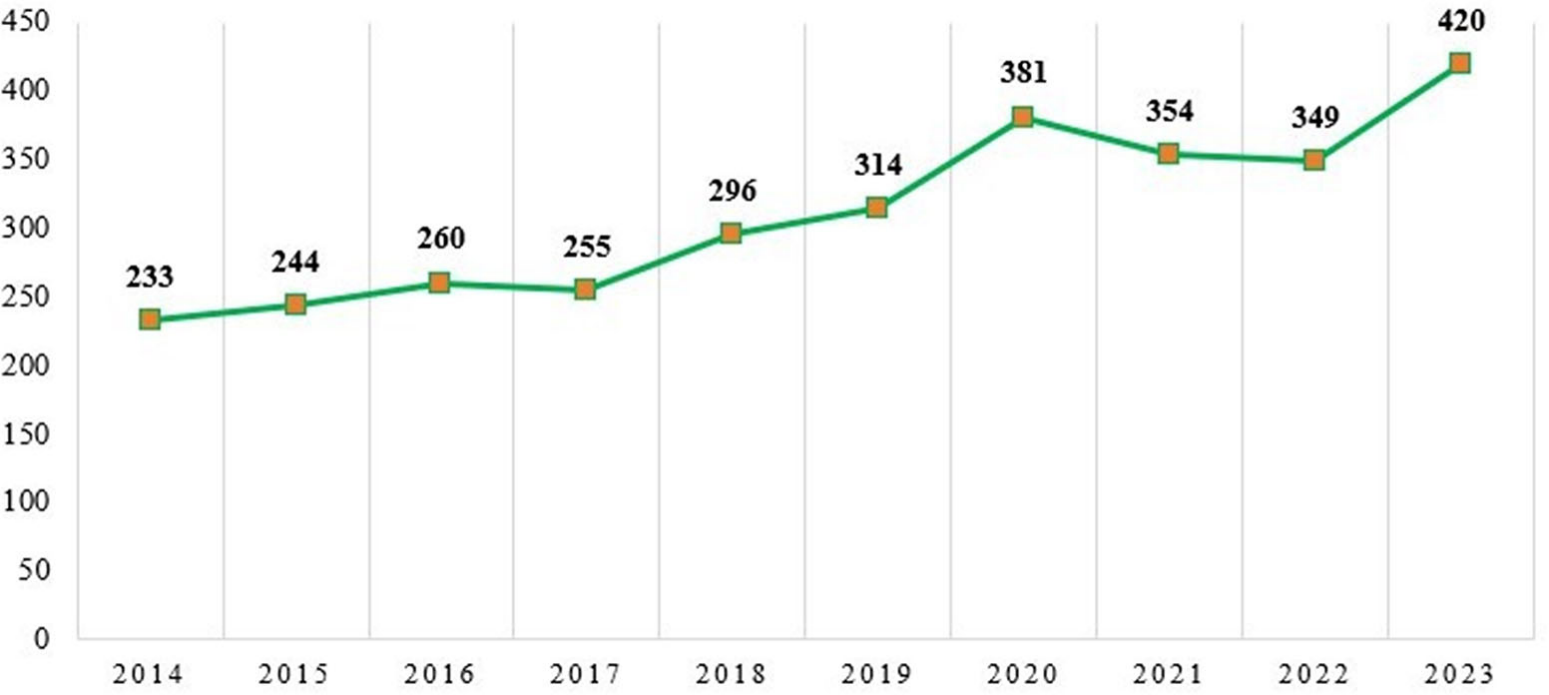
Annual citation trend.

The citation trend for the period 2014–2023 is shown in Figure 4. The citation patterns of these researchers have significantly changed. The year 2023 had the most citations (420), followed by 2020 with 381 citations, whereas 2014, with only 233 articles, witnessed the fewest citations.

### Impact of citations

This scientometric study took all scholarly articles published between 2014 and 2023 on “motivation” and “job satisfaction.” Citations of worthy articles had a significant impact on research. The total number of citations and their average length from 2014 are listed in Table 3.

**Table 3.** Citation count.

Year	Mean TCPerArt	N	Mean TCPerYear	CitableYears
2014	35.01	233	3.18	11
2015	26.08	244	2.61	10
2016	22.65	260	2.52	9
2017	39.73	255	4.97	8
2018	17.49	296	2.50	7
2019	12.90	314	2.15	6
2020	12.76	381	2.55	5
2021	10.79	354	2.70	4
2022	5.92	349	1.97	3
2023	2.58	420	1.29	2

N=Total Number of Citation.

### Major source title of publications

**Figure 5.**
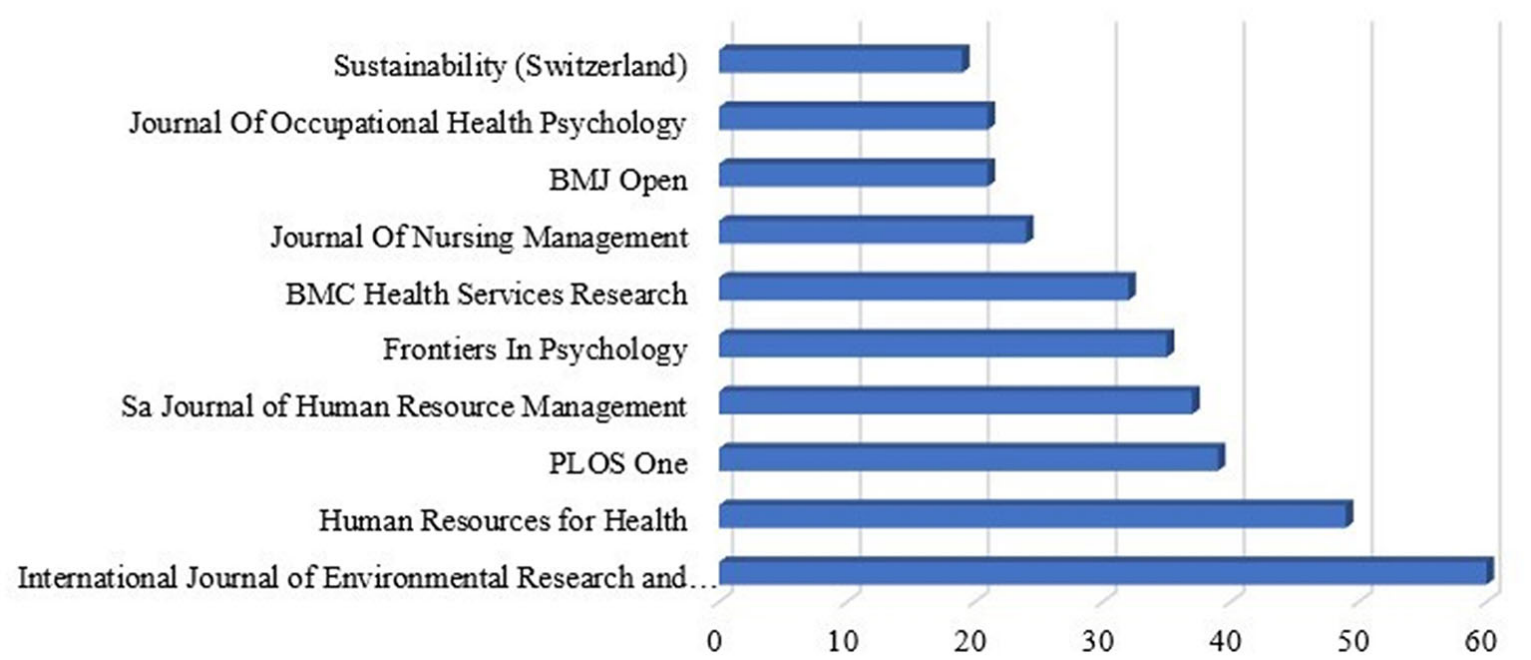
Most relevant sources.

Figure 5 shows that publication shares the most productive sources that produce complete documents (3106) in the area of “Motivation” and “Job Satisfaction.” The scientific literature on motivation and job satisfaction has spread over 1648 different source journals, conference proceedings, and books, among others.

It is found that the “International Journal of Environmental Research and Public Health” leads the table for 2023, followed by “Human Resources for Health” and “PLOS One”. The numbers and their percentages according to the total number of publications are depicted in Table 4.

**Table 4.** Top cited journals.

Sources title	Articles	Citation	2023 Impact Factor (IF)	SJR
International Journal of Environmental Research and Public Health	60	600	4.614	0.808
Human Resources for Health	49	1689	3.9	1.577
PLOS One	39	627	2.9	0.839
SA Journal of Human Resource Management	37	236	1.2	0.297
Frontiers In Psychology	35	999	2.6	0.800
BMC Health Services Research	32	1013	2.7	1.029
Journal of Nursing Management	24	554	3.7	1.485
BMJ Open	21	366	2.4	0.971
Journal of Occupational Health Psychology	21	5016	5.9	2.169
Sustainability (Switzerland)	19	586	3.3	0.672

N (Total Number of Sources) =1648.

**Figure 6.**
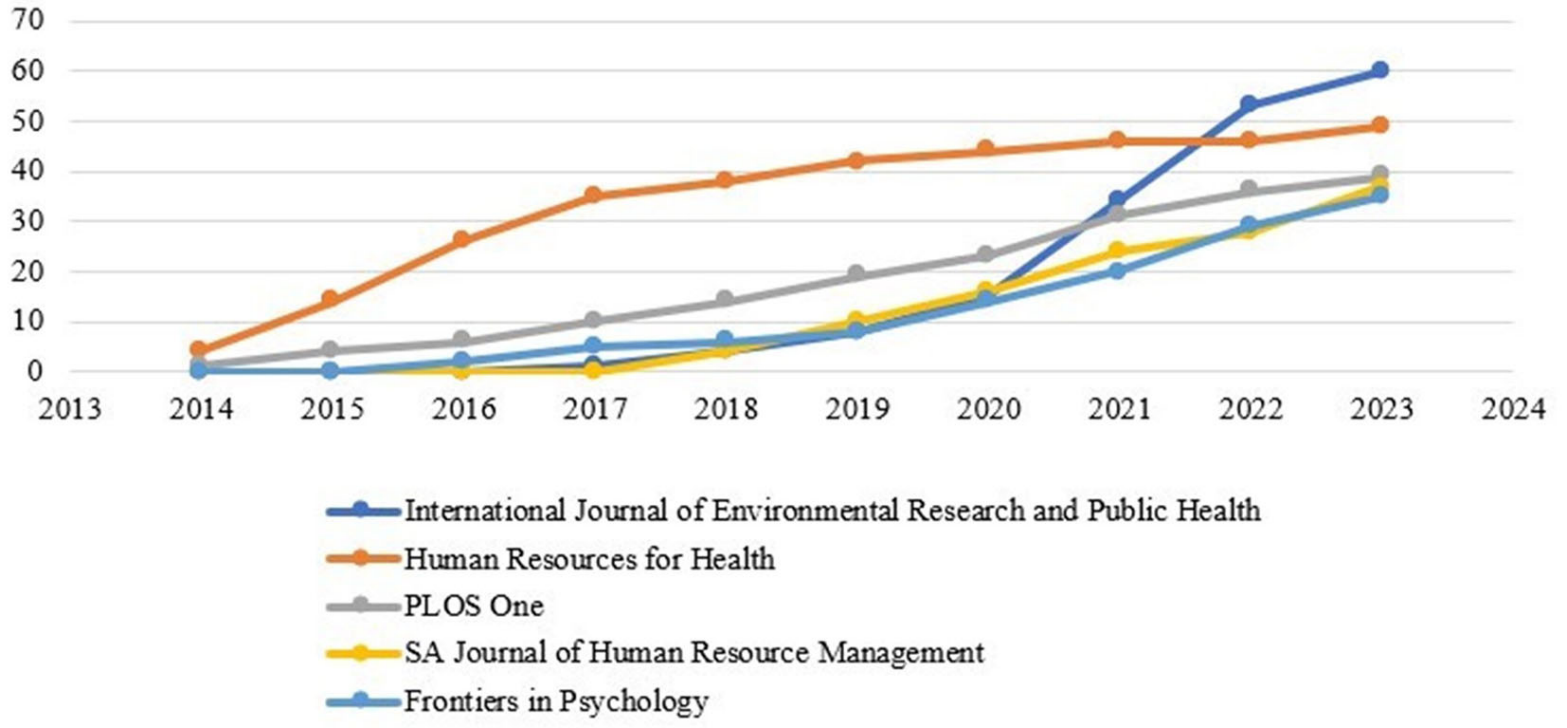
Source production over time.

The production of the source over time is shown in Figure 6. For a graphical representation, the top five sources were combined. Production over time is depicted in the figure, indicating that the sources are progressively increasing their research productivity.

Table 5 presents the cumulative number of publications onthe sources. In 2017, the scientific output of the “International Journal of Environmental Research and Public Health” was marked by just one article and continued to grow steadily. In 2019, the output nearly doubled. In addition, it has doubled in 2020 and 2021. The same number of outputs is produced by the second source, “Human Resources for Health.” Between 2019 and 2023, the output exceeded 40. On the contrary, the “PLOS One” also produces its scholarly production on an average range. Since 2018, the “SA Journal of Human Resource Management” has produced scientific work and consistently published the latest findings of various studies on motivation and job satisfaction. The journal entitled “Frontiers in Psychology” published papers on motivation and job satisfaction in 2016 and reached 35 by the year 2023.

**Table 5.** Major source titles.

Year	International journal of environmental research and public health	Human resources for health	PLOS One	SA journal of human resource management	Frontiers in psychology
2023	60	49	39	37	35
2022	53	46	36	28	29
2021	34	46	31	24	20
2020	15	44	23	16	14
2019	8	42	19	10	8
2018	4	38	14	4	6
2017	1	35	10	0	5
2016	0	26	6	0	2
2015	0	14	4	0	0
2014	0	4	1	0	0
Total	175	344	183	119	119

Table 5 shows that the source journal, Human Resources for Health has published the maximum number of articles on motivation and job satisfactions (344 articles) followed by PLOS One (183 articles), and International Journal of Environmental Research and Public Health (175 articles) from 2014 to 2023 while, ‘SA Journal of Human Resource Management’ and ‘Frontiers in Psychology’ have published an equal number of 119 each from 2014 to 2023.

### Most prolific authors

**Figure 7.**
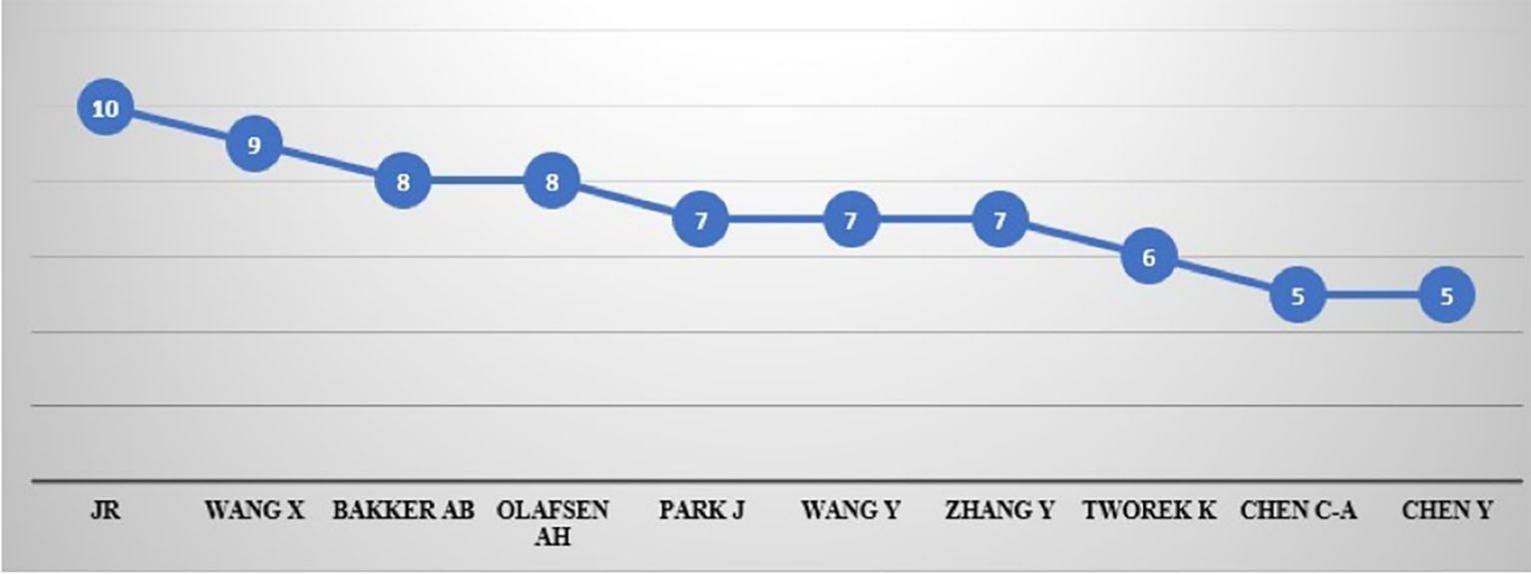
Author's contribution.

Table 6 and Figure 7 show the top 10 authors with 72 articles over the years with 10 (0.32%) publications. JR leads the scientific literature on productivity, followed by Wang, X. with 9 (0.29%), Bakker, A.B., and Olafsen, A.H. with 8 (0.26%), Park, J., Wang, Y., and Zhang, Y. with 7 (0.23%), Tworek, K. with six (0.19%) publications, Chen, C-A. and Chen, Y.B. with 5 (0.16%) each.

**Table 6.** Prolific authors.

Authors	Articles	Percentage (%)
JR	10	0.32
WANG X	9	0.29
BAKKER AB	8	0.26
OLAFSEN AH	8	0.26
PARK J	7	0.23
WANG Y	7	0.23
ZHANG Y	7	0.23
TWOREK K	6	0.19
CHEN C-A	5	0.16
CHEN Y	5	0.16

The most successful authors relied on their indices and publications. Well-known authors and their indices are listed in Table 7. The top 10 authors, according to the maximum H-index, are listed in Table 7. With a maximum H-Index of 9, G-Index of 10, and M-Index of 0.818, as well as 10 publications with 996 citations, author “JR” is reported to have a significant contribution to research followed by Bakker, A. who also has the same H & G index and eight publications. There were 3777 citations in total. This demonstrates an outstanding scientific contribution. Only two of the 10 authors have more than 3000 citations, meaning that Bakker and Demerouti have average article citations of over 450 and 600, respectively. The prolific authors who produced their highest outputs and received more citations in 2014 and 2015 are highlighted in Table 7.

**Table 7.** Authors metric.

Element	H Index	G Index	M Index	TC	NP	PY Start
JR	9	10	0.818	996	10	2014
BAKKER AB	8	8	0.727	3777	8	2014
OLAFSEN AH	7	8	0.636	1717	8	2014
CHEN C-A	5	5	0.455	111	5	2014
DEMEROUTI E	5	5	0.455	3384	5	2014
FERNET C	5	5	0.5	237	5	2015
GOLDACRE MJ	5	5	0.5	139	5	2015
HITKA M	5	5	0.5	101	5	2015
LAMBERT TW	5	5	0.5	142	5	2015
LIU X	5	5	0.455	133	5	2014

### Highly productive countries

**Figure 8.**
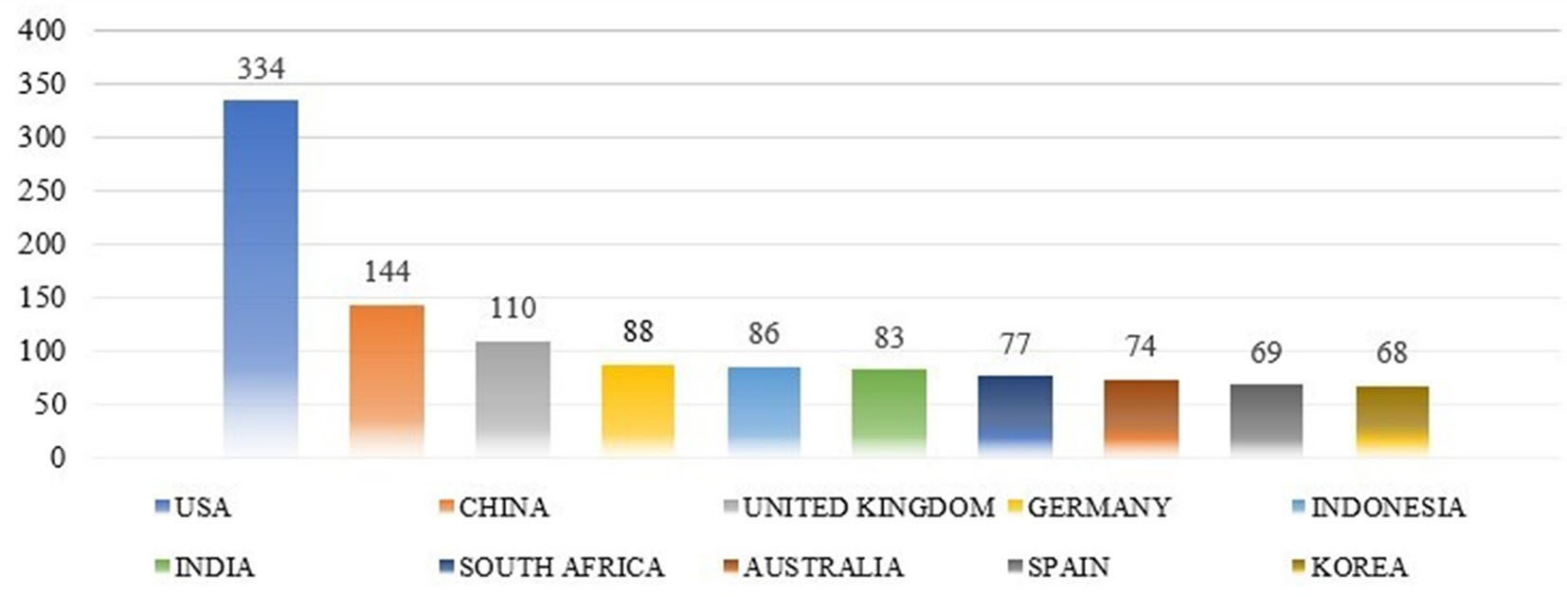
Productive countries.

Figure 8 shows the proportion of 3106 publications from highly productive countries. The researchers determined the top 10 nations based on the total number of citations and the research productivity of the authors.

The highest number of articles originated in the USA States 334 (10.75%), followed by China 144 (4.63%), the UK Kingdom 110 (3.54%), Germany 88 (2.83%), Indonesia 86 (2.77%), India 83 (2.67%), South Africa 77 (2.48%), Australia 74 (2.38%), Spain 69 (2.22%), and Korea 68 (2.19%). The majority of academic publications on “Job Satisfaction” and “motivation” originate from developed countries, such as the USA, China, and the UK.

**Figure 9.**
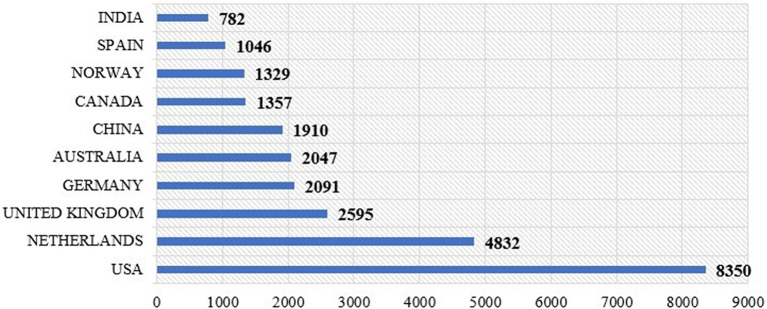
Citation among countries.

Figure 9 shows the number of citations received by papers from different countries. The USA has the highest number of citations, at 8,350, constituting 20.35% of the total citations. This was followed by the Netherlands, with 4,832 citations (11.78%); the United Kingdom, 2,585 (6.30%); Germany, 2,091 (5.10%); Australia, 2,047 (4.99%); China, 1,910 (4.66%); Canada, 1,352 (3.30%); Norway, 1,329 (3.24%); Spain, 1,046 (2.55%); and India, 782 (1.91%).

**Figure 10.**
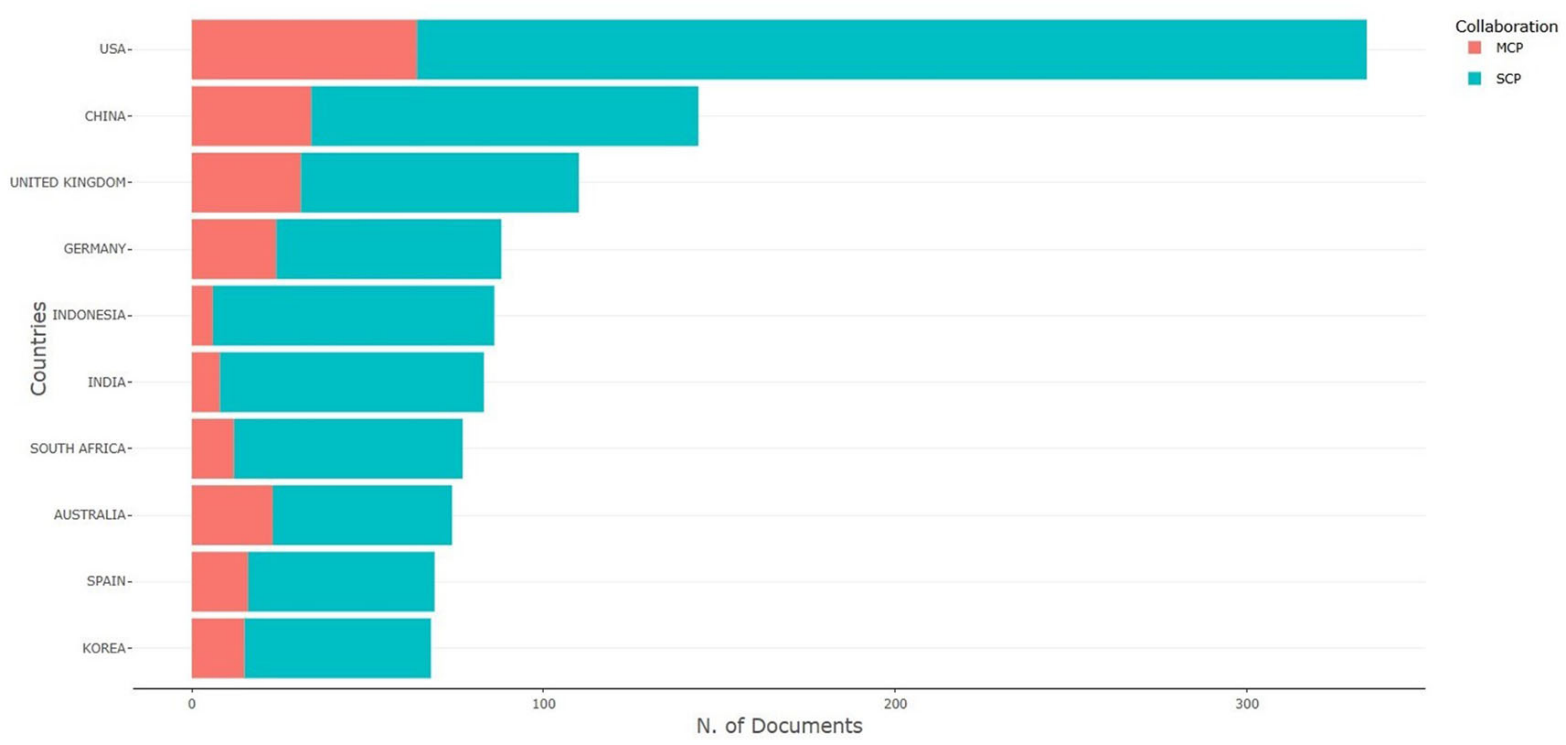
Author's production.

Figure 10 shows the multiple-country publication (MCP) and single-country publication (SCP) of all publications. The publication indicates that the USA ranks first with SCP 270 and MCP 64, signifying that there are 270 (80.83%) single-author publications in the USA, while collaborative works involving the authors of the USA amount to 64 (19.17%). China published 144 scholarly outputs, of which 110 (76.39%) were single-author publications, while only 34 (23.61%) were multiple-author publications. The United Kingdom has 110 publications, of which 79 (71.82%) are single-author works and 31 (28.18%) are collaborative. Table 8 presents the MCP and SCP scores categorized by country. All the countries exhibited the highest number of single-author publications from 2014 to 2023. The article entitled, “Subjective career success: A meta-analytic review” by Ng TWH, published in 2014 with 305 scopus citations, which is also considered as an impactful article, occupies the 10 ^th^ position.

**Table 8.** Country scholarly output as per SCP & MCP.

Country	Articles	SCP	MCP	Frequency	MCP Ratio
USA	334	270	64	0.108	0.192
CHINA	144	110	34	0.046	0.236
UNITED KINGDOM	110	79	31	0.035	0.282
GERMANY	88	64	24	0.028	0.273
INDONESIA	86	80	6	0.028	0.07
INDIA	83	75	8	0.027	0.096
SOUTH AFRICA	77	65	12	0.025	0.156
AUSTRALIA	74	51	23	0.024	0.311
SPAIN	69	53	16	0.022	0.232
KOREA	68	53	15	0.022	0.221

### Highly cited articles

Table 9 shows that the article entitled, “Job demands-resources theory: Taking stock and looking forward” by Bakker AB published in the year 2017 has received the highest number of 2912 citations followed by the article entitled, Self-Determination Theory in Work Organizations: The State of a Science by Deci EL, published in 2017 (14 57 citations); and Intrinsic motivation and extrinsic incentives jointly predict performance: a 40-year meta-analysis by Cerasoli Cp published in 2014 (1121 citations).

**Table 9.** Highly cited articles.

Rank	Paper	Title	Total citations	TC per year	Google scholar citation
1	Bakker AB, 2017	Job demands-resources theory: Taking stock and looking forward	2912	323.56	6820
2	Deci EL, 2017	Self-Determination Theory in Work Organizations: The State of a Science	1457	161.89	2250
3	Cerasoli Cp, 2014	Intrinsic motivation and extrinsic incentives jointly predict performance: a 40-year meta-analysis	1121	93.42	3062
4	Martin R, 2016	Leader–Member Exchange (LMX) and Performance: A Meta-Analytic Review	506	50.60	767
5	Parker SK, 2014	Beyond motivation: job and work design for development, health, ambidexterity, and more	437	36.42	1003
6	Sonnentag S, 2017	Advances in recovery research: What have we learned? What should be done next?	398	44.22	759
7	Okuyama A, 2014	Speaking up for patient safety by hospital-based health care professionals: a literature review	337	28.08	380
8	Judge TA, 2017	Job attitudes, job satisfaction, and job affect: A century of continuity and of change	333	37.00	613
9	Miraglia M, 2016	Going to work ill: A meta-analysis of the correlates of presenteeism and a dual-path model	314	31.40	476
10	Ng TWH, 2014	Subjective career success: A meta-analytic review	305	25.42	683

### The co-authorship network among countries

**Figure 11.**
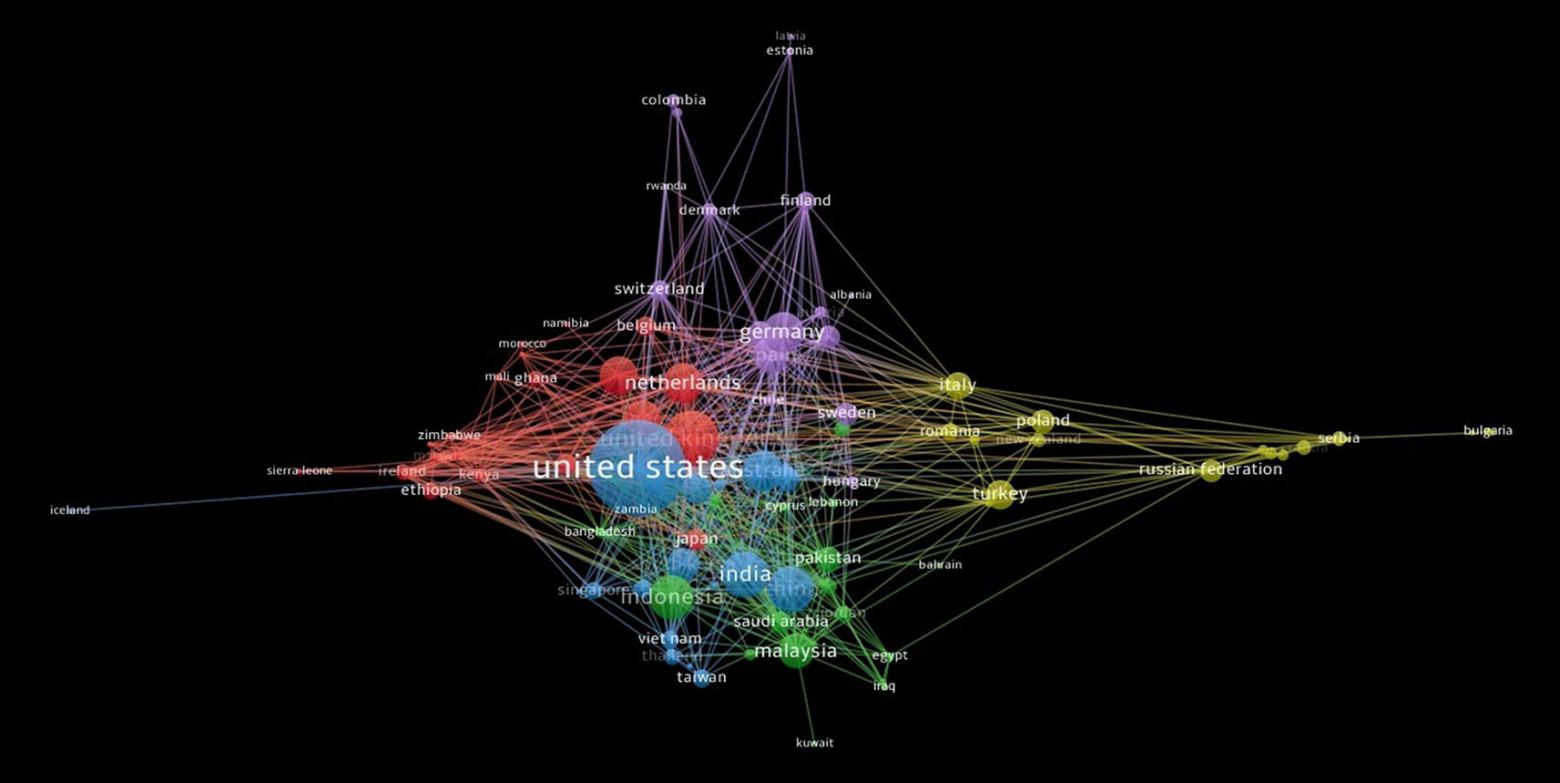
Co-authorship pattern among countries.

Figure 11 shows co-authorship networks across countries and regions. In terms of node size, the United States has the most extensive research on this topic. China, the Netherlands, Germany, and India have strong co-authorship relationships with others. The 98 countries that met the threshold (Threshold = Minimum Number of Publications three Per Country) collaborated on the research. Five clusters are formed from the countries; the largest cluster is the United Kingdom, and the first cluster, symbolized by the red, includes twenty-three other countries. The second cluster, which includes 21 countries and is tinted green, includes Malaysia and Indonesia. Australia and the United States are among the 19 countries that make up the third cluster, which is blue. The United States works with all the cluster nations. The fourth cluster consists of 18 countries, with Italian, Romanian, Polish, and Turkish countries indicated in yellow. Seventeen purple-coloredcountries are dispersed over Germany, Spain, Finland, and other countries in the fifth and final clusters.

### Co-citation network analysis

Co-citation is a term used to describe how frequently two previous works are cited jointly in later works. It is an effective method for mapping the scientific intellectual structure, since co-citation trends over time might reveal insights into the process of the area of expertise. Author co-citations and their intellectual relationships are always reflected in the scientific literature. Therefore, by analyzing the author’s co-citation, influential scholars in the field of both “Motivation” and “Job Satisfaction.” Of the 159529 authors, only 1635 authors met the threshold (Threshold: Minimum Number of Citation = 20). The network contains 532352 links with 2920868 total link strength.

**Figure 12.**
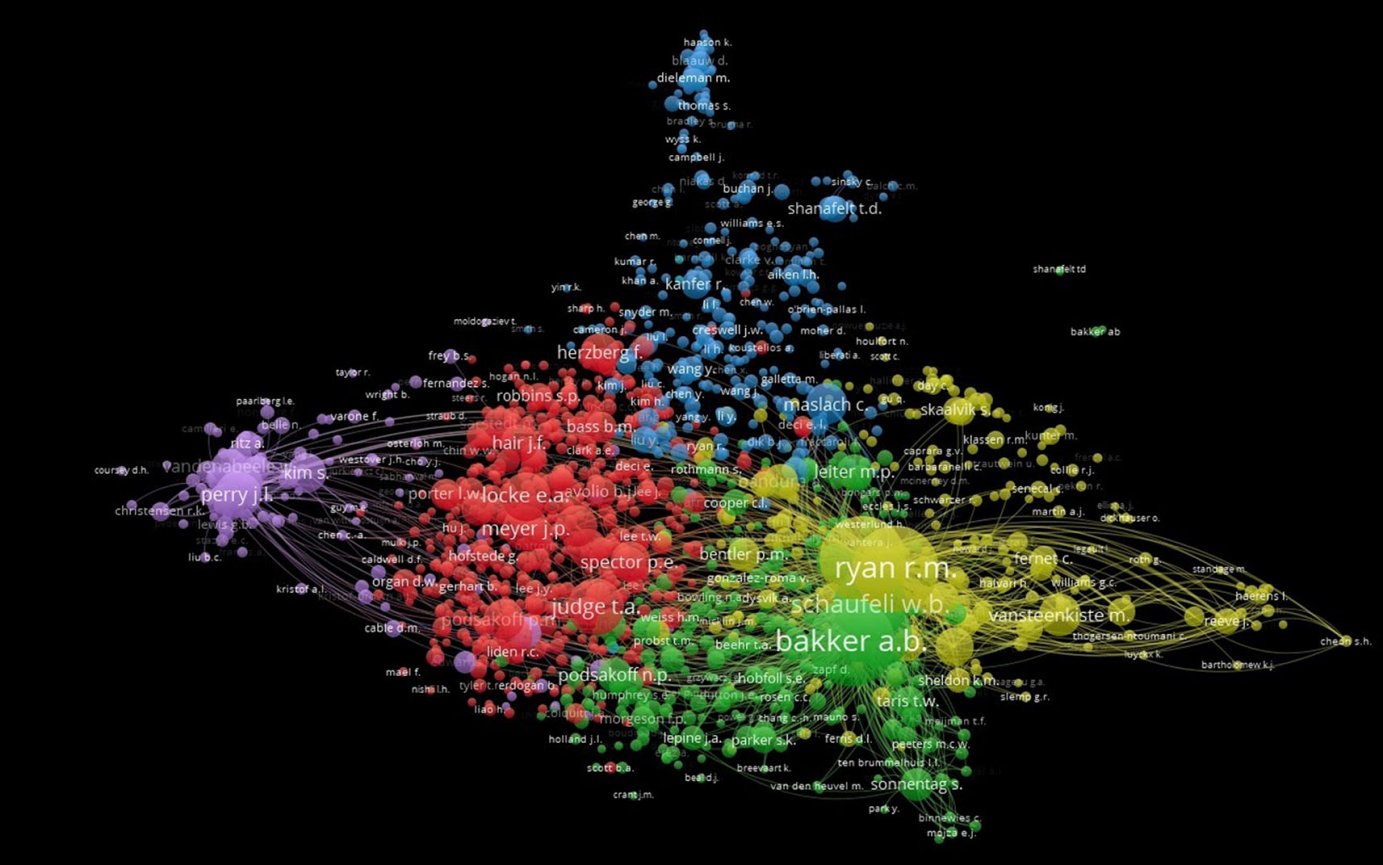
Co-citation analysis.

Figure 12 shows the authors’ co-citation network. Six clusters are represented by six distinct colors. Cluster 1 is represented in red, comprising 630 items; Cluster 2 is indicated in green, containing 318 items; Cluster 3 is denoted in blue, with 316 items; Cluster 4 is marked in yellow, including 250 authors; Cluster 5 is depicted in purple, with a total of 118 authors; and Cluster 6 is represented bythree authors. The most cited author is Rayan, R.M., and Bakker, A.B. is the most prominent author in the network owing to substantial academic contributions in the fields of “Motivation” and “Job Satisfaction”.

### Keyword analysis

The investigation was intensified through a detailed examination of keyword-explosion detection. Keywords encompass the essential material of a research piece, representing authors’ scholarly insights and perspectives. A significant concept can be discerned through a keyword co-occurrence network within a certain knowledge domain over a designated time frame. The evolution of a network can elucidate the progression of the research domain over time.

**Figure 13.**
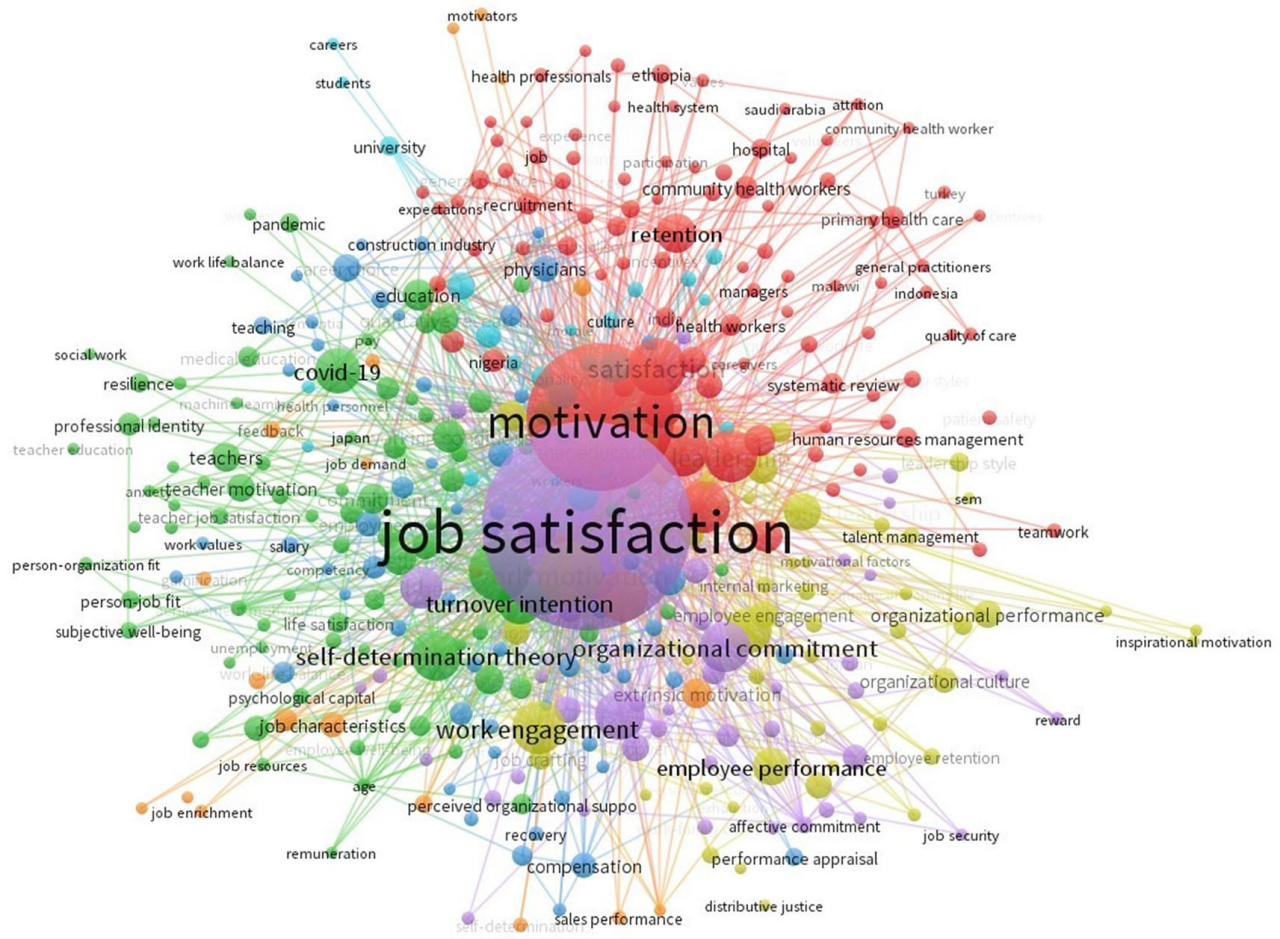
Keyword analysis.

The keyword analysis in Figure 13 shows the most frequently used keywords on Job Satisfaction- and motivation-related papers. The frequency of keyword occurrences correlates positively with the size of associated keyword labels. The frequency of keyword occurrences significantly contributed to achieving the research objectives. In addition to Job Satisfaction, the terms Motivation, Satisfaction, Turnover Intention, and Organizational Management were also prevalent keywords. These themes hold pivotal positions in the Job Satisfaction.

### Key findings

• The study found that the highest number of articles on motivation and job satisfaction was published in 2023 (420 articles), and the lowest number of articles was published in 2014 (233 articles).
• In terms of citations to published articles on motivation and job satisfaction, the year 2023 had the most citations (420), followed by 2020 with 381 citations, while 2014 with only 233 articles witnessed the fewest citations.
• It is found that the source journal, Human Resources for Health has published the maximum number of articles on motivation and job satisfactions (344 articles) followed by PLOS One (183 articles), and International Journal of Environmental Research and Public Health (175 articles) from 2014 to 2023 while, ‘SA Journal of Human Resource Management’ and ‘Frontiers in Psychology’ have published an equal number of 119 each from 2014 to 2023.
• Among the most prolific authors who have contributed articles in the field of motivation and job satisfaction, JR leads the table, followed by Wang, X. with 9 (0.29%), Bakker, A.B., and Olafsen, A.H. with 8 (0.26%), Park, J., Wang, Y., and Zhang, Y. with 7 (0.23%), Tworek, K. with 6 (0.19%) publications, Chen, C-A. and Chen, Y.B. with 5 (0.16%) each.
• In terms of country productivity, the highest number of articles originated in the USA States 334 (10.75%), followed by China 144 (4.63%), the UK 110 (3.54%), Germany 88 (2.83%), Indonesia 86 (2.77%), India 83 (2.67%), South Africa 77 (2.48%), Australia 74 (2.38%), Spain 69 (2.22%), and Korea 68 (2.19%). The majority of academic publications on “Job Satisfaction” and “motivation” originate from developed countries such as the USA, China, and the UK.
• In terms of highly cited articles in the area of motivation and job satisfaction, it was found that the article entitled, “Job demands-resources theory: Taking stock and looking forward” by Bakker AB published in the year 2017 has received the highest number of 2912 citations followed by the article entitled, “Self-Determination Theory in Work Organizations: The State of a Science” by Deci EL, published in 2017 (14 57 citations); and “Intrinsic motivation and extrinsic incentives jointly predict performance: a 40-year meta-analysis” by Cerasoli Cp published in 2014 (1121 citations).

## Discussion

Scientometric analysis shows a strong and quickly growing body of literature over the last ten years that focuses on Job Satisfaction and Motivation ( Keith et al., 2024). We examined research outputs in Motivation and Job Satisfaction during the last decade to discern publishing trends, growth, prolific writers, contributing institutions, collaboration patterns, keyword occurrence, and the co-citation patterns of authors. It is found that the research productivity on motivation and job satisfaction has risen from 2014 to 2023. The region has been the primary focus of scholars in the subjects of Job Satisfaction and Motivation for some years, as evidenced by the publication of 3,106 articles during the last decade. The predominant trend from 2020 to 2023 indicates that Job Satisfaction and Motivation have become the subjects of rapidly published research. The recent surge in Job Satisfaction research is attributable to various factors, including the utilization of secondary data and inaccessibility of study samples. Various forms of “HRM” study were disseminated by the writers in the USA. The most favored categories were the publications of systematic reviews and literature reviews. A literature review is the most fundamental and essential element of any research article, thesis, or dissertation. Researchers have frequently published literature reviews as research publications. The findings indicate a significant increase in publications, from 233 in 2014 to 420 in 2023, with an annual growth rate of 6.77%. Citations also rose markedly in the domains of “Job Satisfaction” and “Motivation”. The top three articles received over 1,000 citations annually, which underscores the quality of research, as pointed out by one of studies of Yumnam et al. (2024). The countries with the highest levels of engagementand the strongest co-authorshipare the United States, the United Kingdom, Australia, and Germany. The co-authorship map shows that encouraging collaborative platforms can result in increased productivity and impact, directing future research directions and influencing funding and policy decisions to support collaborative and impactful research output. This highlights the significance of leveraging HRM research and fostering collaboration to improve the dissemination and impact of research. Moreover, the co-cited author network analyzes some highly productive authors and co-networks. Ryan and Bakkerare the most productive authors in the field of Job Satisfaction and Motivation research in the recent past. The findings of this study add niceties to the corpus of existing literature.

### Scope for future study

This study provides a solid platform for future research in this area. Scholars may undertake future research in the following ways:

• A comparative bibliometric study of motivation and job satisfaction reflected in Scopus and the Web of Science may be undertaken.
• A bibliometric study of different management topics may be conducted in light of the findings of this study.
• A bibliometric study of published literature on motivation and job satisfaction for a different range of time periods, including the published literature of 2024, may be taken into the purview of research.

## Conclusion

The evolution of publications on “Motivation” and “Job Satisfaction” from 2014 to 2023 showed a steady growth rate, despite annual variations over the previous ten years. Research on job satisfaction and motivation has advanced through the work of authors from the USA. We mapped the entire research structure and features of job satisfaction research over the past decade. This was accomplished by examining the research topics of motivation and job satisfaction in a corpus of recent publications. The most prolific authors on the topic of job satisfaction and motivation, “JR,” “Wang, X.,” and “Bakker, A.B.”, are either from the United States or the United Kingdom. The most productive author, JR, wrote 10 articles during the year with 9 H-indexes and 10 G-indexes, indicating high continuity in research. Bakker has eight publications, but most of the citations come from him alone. Document co-citation analysis was used to identify and group documents from Job Satisfaction and Motivation studies with high co-citation strength into six clusters. Each cluster indicates a distinct author, and Ryan, R.M. and Bakker, A.B. collaborate with other authors the most; this indicates the largest number of times these two authors are cited simultaneously. Finally, a historical analysis revealed a gradual rise in scientific output on “Job Satisfaction” and “motivation” from 2014 to 2023. Overall, the study’s findings show that research productivity in the Human Resource Management literature is increasing, which seems to be a healthy trend.

## Ethical considerations

Ethical approval and consent were not required.

## Data Availability

No data associated with this article. The data for this article consists of bibliographic references, which are included in the references section.
