# Supplementary material for: Research Trends on Motivation and Job Satisfaction: A Scientometric Analysis
Source: F1000Res. 2026 Jan 21;14:703. Originally published 2025 Jul 17. [Version 2] doi: 10.12688/f1000research.165866.2 (PMC12887373; doi:10.12688/f1000research.165866.2)
Supplement: Peer review history

# Reviewer report: Reviewer response for version 2

**DOI:** 10.5256/f1000research.195204.r455122

**Published:** 2026-02-18

**Authors:** Dinh-Hai Luong

**Affiliations:** 1 Vietnam National University of Educational Sciences, Hanoi, Hanoi, Vietnam

Overall, the manuscript follows the journal’s required structure. The authors employ a systematic data collection process and draw on a range of analytical methods and software tools. However, the manuscript would benefit from a clearer and more transparent account of the data collection and screening procedures. In addition, I encourage the authors to consider incorporating a qualitative content analysis to better interpret the descriptive results and to strengthen the study’s overall contribution. My specific comments are as follows:

1. Please reorganize the Abstract to clearly state the research aims, the main findings, and the key theoretical and practical implications. The authors may consider (i) specifying the number of documents collected in the Methods, (ii) moving key findings currently presented in the Conclusion into the Results section, and (iii) expanding the Conclusion to include clearer insights and actionable recommendations.
2. Please ensure consistency in how the research context is presented. In particular, reconcile the statement that “There are significant numbers of studies conducted on Herzberg motivation theory but only a few studies have been conducted in this direction” with the later claim that “Many studies have been published on motivation and job satisfaction” in the Introduction.
3. Please review and restructure the literature review to focus more explicitly on prior studies related to job satisfaction and motivation. The discussion should be coherent, logically sequenced, and directly aligned with the study’s scope and research aims.
4. Although “bibliometrics” and “scientometrics” are sometimes used interchangeably, please select one term and apply it consistently throughout the manuscript.
5. In the Methods section, please provide a clearer account of the data collection and screening process, including specific inclusion/exclusion (filtration) criteria and the data analysis techniques used. It should also be stated explicitly that the findings are based only on documents directly relevant to job satisfaction and motivation. Strengthening methodological transparency will improve the credibility and interpretability of the study’s results.
6. As the current results are primarily descriptive, the Discussion remains somewhat limited. The authors may consider adding a qualitative content analysis of the most salient findings to deepen interpretation and strengthen the manuscript’s theoretical and practical contributions.

I hope these suggestions help clarify my concerns and support the authors in further improving the manuscript.

Is the work clearly and accurately presented and does it cite the current literature?

Partly

If applicable, is the statistical analysis and its interpretation appropriate?

Yes

Are all the source data underlying the results available to ensure full reproducibility?

Yes

Is the study design appropriate and is the work technically sound?

Yes

Are the conclusions drawn adequately supported by the results?

Yes

Are sufficient details of methods and analysis provided to allow replication by others?

Partly

Reviewer Expertise:

open and distance learning, e-learning, information communication technology in education, library and information science, open educational resources.

I confirm that I have read this submission and believe that I have an appropriate level of expertise to confirm that it is of an acceptable scientific standard, however I have significant reservations, as outlined above.

# Reviewer report: Reviewer response for version 2

**DOI:** 10.5256/f1000research.195204.r451938

**Published:** 2026-02-09

**Authors:** Ferda Alper Ay

**Affiliations:** 1 Sivas Cumhuriyet University, Sivas, Turkey

The authors have further enhanced the article. Congratulations.

Is the work clearly and accurately presented and does it cite the current literature?

Partly

If applicable, is the statistical analysis and its interpretation appropriate?

Yes

Are all the source data underlying the results available to ensure full reproducibility?

Yes

Is the study design appropriate and is the work technically sound?

Yes

Are the conclusions drawn adequately supported by the results?

Partly

Are sufficient details of methods and analysis provided to allow replication by others?

Yes

Reviewer Expertise:

Management and strategy, management and organization, organizational psychology, human resources management, work motivation, organizational culture, nepotism, mobbing, leadership, organizational citizenship behavior, UPB, whistleblowing, malicious supervision, inefficient work behaviors, and health management.

I confirm that I have read this submission and believe that I have an appropriate level of expertise to confirm that it is of an acceptable scientific standard.

# Response

**Published:** 2026-02-09

**Authors:** Dr. Dillip K Swain

**Affiliations:** Library & Information Science, Kalinga Institute of Industrial Technology, Bhubaneswar, Odisha, India

The meticulous review is highly appreciated. Thanks and regards

# Reviewer report: Reviewer response for version 1

**DOI:** 10.5256/f1000research.182612.r439224

**Published:** 2026-01-10

**Authors:** Ferda Alper Ay

**Affiliations:** 1 Sivas Cumhuriyet University, Sivas, Turkey

Dear Editor and Authors,

 This study is a robust bibliometric analysis of 10 years of scientific research on motivation and job satisfaction, spanning 2014-2023. The analysis is detailed. However, the article could be further improved. My suggestions for improving the article are as follows:

 1. The structured abstract mentions the methodology in the background. Add background information to the abstract.

 2. “Hertzberg posits that employees’ level of job satisfaction is truly influenced by both internal and external factors in their working ambience. Job satisfaction and motivation are related to each other; if one is affected, the other will also be affected. Few studies have been conducted on this topic.”

 Add a footnote for Hertzberg in this sentence. Also, there are numerous studies on this topic, and a significant portion of motivation and job satisfaction research utilizes Hertzberg.

 This sentence (“Few studies have been conducted on this topic.”) is incorrectly worded or does not seem appropriate. It should be rechecked.

 3. Check the following statement after the definition of motivation: “Motivation is a psychological concept that cannot be applied to employees.”

 This statement can lead to misunderstandings, as if motivation were entirely internal and unmanageable. Therefore, it contradicts modern theories such as Herzberg's Two-Factor Theory.

 Please refer to the following source on this topic:

 -"Motivation is influenced by internal and external sources and basic psychological needs. Managers can both increase employees' external motivation by regulating external conditions and create a favorable environment to foster internal motivation. Therefore, motivation fundamentally stems from the individual's internal processes; however, managers can significantly influence employees' motivation levels by providing appropriate external conditions." Please refer to source 1: Karakaya, A. ve Ay, F.A. (2007), Çalışanların Motivasyonunu Etkileyen Faktörler: Sağlık Çalışanlarına Yönelik Bir Araştırma, C.Ü. Sosyal Bilimler Dergisi, Cilt : 31 No:1, ss. 55- 67.

 4. Literature Review

 This section presents information on methods used in previous research, such as Biblioshiny, VOSviewer, and BibExcel. However, before this information, the study should include a literature review, primarily focusing on motivation and job satisfaction.

 5. Checking the sources is necessary. For example, check the following information in the article:

 “Kannan, Kulandai, & Ramachandran (2024) in the bibliometric analysis revealed key factors associated with motivation, job satisfaction, and retention of nurses in their respective working places. Moreover, the study significantly contributes to the understanding of nurse job satisfaction, retention dynamics, and assorted facets of banking on the findings of earlier studies.”

 Upon examination of this research (Kannan, Kulandai, & Ramachandran (2024)), it was found that it did not include a bibliometric analysis, although this was mentioned in your explanations.

 6. In line with the purpose of the study, only scientific studies were reviewed. Explain the relationship between motivation and job satisfaction, supported by the literature, for high impact.

 7. Table 2 appears to be missing data for other years.

 8. The statement regarding data availability can be reiterated. As you stated in the article, “Publication data on job satisfaction, ranging from 2014 to 2023, were extracted from Scopus.” (Your data consists of these studies).

 9. Add limitations to your study.

 10. Utilize the most influential articles included in your findings.Support your analysis of motivation and job satisfaction with substantial supporting literature, and add practical implications based on your article.

 Best regards.

Is the work clearly and accurately presented and does it cite the current literature?

Partly

If applicable, is the statistical analysis and its interpretation appropriate?

Yes

Are all the source data underlying the results available to ensure full reproducibility?

Yes

Is the study design appropriate and is the work technically sound?

Yes

Are the conclusions drawn adequately supported by the results?

Partly

Are sufficient details of methods and analysis provided to allow replication by others?

Yes

Reviewer Expertise:

Management and strategy, management and organization, organizational psychology, human resources management, work motivation, organizational culture, nepotism, mobbing, leadership, organizational citizenship behavior, UPB, whistleblowing, malicious supervision, inefficient work behaviors, and health management.

I confirm that I have read this submission and believe that I have an appropriate level of expertise to confirm that it is of an acceptable scientific standard, however I have significant reservations, as outlined above.

## References

[1] Mohammed Alshmemri, Lina Shahwan-Akl and Phillip Maude. Herzberg’s Two-Factor Theory. Life Sci J 2017;14(5):12-16]. ISSN: 1097-8135 (Print) / ISSN: 2372-613X (Online). http://www.lifesciencesite.com. 3. doi:10.7537/marslsj140517.03.

[2] : Examining Public Service Motivation’s Impact on Organizational Commitment: Focusing on Moderating Roles of Hygiene and Motivation Factors. *Behavioral Sciences* .2024;14(6) : 10.3390/bs14060476 10.3390/bs14060476 PMC1120126538920808

[3] : Motivation and job satisfaction. *Management Decision* .1998;36(4) : 10.1108/00251749810211027 226-231 10.1108/00251749810211027

[4] : Job Satisfaction in The Public Service. *Public Management Review* .2011;13(5) : 10.1080/14719037.2010.532959 731-751 10.1080/14719037.2010.532959

# Response

**Published:** 2026-01-12

**Authors:** Dr. Dillip K Swain

**Affiliations:** Library & Information Science, Kalinga Institute of Industrial Technology, Bhubaneswar, Odisha, India

Sl No

 Comments

 Compliance

 1

 The structured abstract mentions the methodology in the background. Add background information to the abstract.

 Thanks a lot to the esteemed reviewer.

 2

 “Hertzberg posits that employees’ level of job satisfaction is truly influenced by both internal and external factors in their working ambience. Job satisfaction and motivation are related to each other; if one is affected, the other will also be affected. Few studies have been conducted on this topic.”

 Add a footnote for Hertzberg in this sentence. Also, there are numerous studies on this topic, and a significant portion of motivation and job satisfaction research utilizes Hertzberg.

 This sentence (“Few studies have been conducted on this topic.”) is incorrectly worded or does not seem appropriate. It should be rechecked.

 Thanks a lot to the esteemed reviewer. We have expanded the discussion. Instead of a footnote we have added the detailed reference (Herzberg, F. (1959)) in the reference list

 3

 Check the following statement after the definition of motivation: “Motivation is a psychological concept that cannot be applied to employees.”

 This statement can lead to misunderstandings, as if motivation were entirely internal and unmanageable. Therefore, it contradicts modern theories such as Herzberg's Two-Factor Theory.

 Please refer to the following source on this topic:

 -"Motivation is influenced by internal and external sources and basic psychological needs. Managers can both increase employees' external motivation by regulating external conditions and create a favorable environment to foster internal motivation. Therefore, motivation fundamentally stems from the individual's internal processes; however, managers can significantly influence employees' motivation levels by providing appropriate external conditions." Please refer to source 1: Karakaya, A. ve Ay, F.A. (2007), Çalışanların Motivasyonunu Etkileyen Faktörler: Sağlık Çalışanlarına Yönelik Bir Araştırma, C.Ü. Sosyal Bilimler Dergisi, Cilt : 31 No:1, ss. 55- 67.

 Thanks to the esteemed reviewer.

 We have developed the section

 4

 This section presents information on methods used in previous research, such as Biblioshiny, VOSviewer, and BibExcel. However, before this information, the study should include a literature review, primarily focusing on motivation and job satisfaction.

 Ans: Done

 5

 Checking the sources is necessary. For example, check the following information in the article:

 “Kannan, Kulandai, & Ramachandran (2024) in the bibliometric analysis revealed key factors associated with motivation, job satisfaction, and retention of nurses in their respective working places. Moreover, the study significantly contributes to the understanding of nurse job satisfaction, retention dynamics, and assorted facets of banking on the findings of earlier studies.”

 Upon examination of this research (Kannan, Kulandai, & Ramachandran (2024)), it was found that it did not include a bibliometric analysis, although this was mentioned in your explanations.

 Ans: Checked and verified. We have fined tuned the information provided

 6

 In line with the purpose of the study, only scientific studies were reviewed. Explain the relationship between motivation and job satisfaction, supported by the literature, for high impact.

 Ans: Thanks to the esteemed reviewer. The revised paper meets the requirement

 7

 Table 2 appears to be missing data for other years.

 Ans: The publications data in Table 2 are provided in two columns: Column 1: Publications from 2014 to 2018; Colum 2: Publications from 2019 to 2023

 8

 The statement regarding data availability can be reiterated. As you stated in the article, “Publication data on job satisfaction, ranging from 2014 to 2023, were extracted from Scopus.” (Your data consists of these studies).

 Ans: Done

 9

 Add limitations to your study.

 Ans: Thanks for the constructive comments of the esteemed reviewer. We have added this facet

 10

 Utilize the most influential articles included in your findings.Support your analysis of motivation and job satisfaction with substantial supporting literature, and add practical implications based on your article.

 Ans: Thanks for the constructive comments of the esteemed reviewer. We have added this facet

# Reviewer report: Reviewer response for version 1

**DOI:** 10.5256/f1000research.182612.r428084

**Published:** 2025-11-10

**Authors:** Mohammad Qais Rezvani, Deepti Kuhar

**Affiliations:** 1 Kurukshetra University,, Kurukshetra, India; 2 Faculty of Commerce and Management, SRM University - Haryana Campus, Sonipat, Haryana, India

**Review Report**

**Title:** *Research Trends on Motivation and Job Satisfaction: A Scientometric Analysis*

 An informative scientometric summary of research trends on job satisfaction and motivation throughout the past ten years (2014–2023) is given in this paper. The authors properly analyzed publication trends, citation patterns, keyword occurrences, and authorship networks using data taken from the Scopus database and scientometric tools, including VOSviewer and Biblioshiny. The methodological strategy is suitable and fits in nicely with the goals of the study.

 The present paper provides an incisive scientometric analysis of research trends on motivation and job satisfaction over the last 10 years (2014–2023). Using data from the Scopus database and scientometric tools including VOSviewer and Biblioshiny, the authors appropriately examined publication trends, citation patterns, keyword occurrences, and authorship networks.

 The methodological approach is appropriate and aligns closely with the study's objectives.

 However, to strengthen the manuscript, the authors could:

1. Extend the discussion section to analyze the consequences of collaboration patterns by author and country.
2. To more clearly depict the bibliometric linkages, use visual aids like co-authorship or keyword co-occurrence maps.
3. Give more details on the search approach, such as the inclusion/exclusion criteria and the kinds of documents that were examined (such as conference papers, reviews, and articles).
4. Discuss limitations—such as database dependence (Scopus only, not Web of Sciences) and potential bias due to language restrictions—to enhance scientific rigor.
5. Provide suggestions for future HRM study avenues based on the tendencies that have been noticed.

Overall, the study makes a significant contribution to the body of existing literature and is cohesive and well-structured. It could be deemed scientifically sound and ready for indexing, if possible, after the aforementioned changes.

Is the work clearly and accurately presented and does it cite the current literature?

Yes

If applicable, is the statistical analysis and its interpretation appropriate?

Yes

Are all the source data underlying the results available to ensure full reproducibility?

Yes

Is the study design appropriate and is the work technically sound?

Yes

Are the conclusions drawn adequately supported by the results?

Yes

Are sufficient details of methods and analysis provided to allow replication by others?

Yes

Reviewer Expertise:

Human Resources Management, Organizational Behviour

We confirm that we have read this submission and believe that we have an appropriate level of expertise to confirm that it is of an acceptable scientific standard.

# Response

**Published:** 2026-01-12

**Authors:** Dr. Dillip K Swain

**Affiliations:** Library & Information Science, Kalinga Institute of Industrial Technology, Bhubaneswar, Odisha, India

Esteemed reviewer,

 We express sincere thanks to your prudent review and appreciation of the article.

 Best regards
